# The novel ZmTCP7 transcription factor targets AGPase-encoding gene *ZmBt2* to regulate storage starch accumulation in maize

**DOI:** 10.3389/fpls.2022.943050

**Published:** 2022-07-15

**Authors:** Babatope Samuel Ajayo, Yangping Li, Yayun Wang, Chengdong Dai, Lei Gao, Hanmei Liu, Guowu Yu, Junjie Zhang, Yubi Huang, Yufeng Hu

**Affiliations:** ^1^State Key Laboratory of Crop Gene Resource Exploration and Utilization in Southwest China, Chengdu, China; ^2^College of Agronomy, Sichuan Agricultural University, Chengdu, China; ^3^College of Life Science, Sichuan Agricultural University, Ya’an, China

**Keywords:** AGPase, endosperm development, gene transcriptional regulation, TCP protein, starch biosynthesis, Y1H screening system

## Abstract

The process of starch biosynthesis is a major developmental event that affects the final grain yield and quality in maize (*Zea mays* L.), and transcriptional regulation plays a key role in modulating the expression of the main players in the pathway. *ZmBt2*, which encodes the small subunits of AGPase, is a rate-controlling gene of the pathway; however, much remains unknown about its transcriptional regulation. Our earlier study identifies a short functional fragment of *ZmBt2* promoter (394-bp), and further shows it contains multiple putative *cis*-acting regulatory elements, demonstrating that several transcription factors may govern *ZmBt2* expression. Here, we identified a novel TCP transcription factor (TF), ZmTCP7, that interacted with the functional fragment of the *ZmBt2* promoter in a yeast one hybrid screening system. We further showed that ZmTCP7 is a non-autonomous TF targeted to the nucleus and predominantly expressed in maize endosperm. Using promoter deletion analyzes by transient expression in maize endosperm protoplasts combined with electrophoretic mobility shift assays, we found that ZmTCP7 bound to GAACCCCAC elements on the *ZmBt2* promoter to suppress its expression. Transgenic overexpression of *ZmTCP7* in maize caused a significant repression of *ZmBt2* transcription by ~77.58%, resulting in a 21.51% decrease in AGPase activity and a 9.58% reduction in the endosperm starch content of transgenic maize. Moreover, the expressions of *ZmBt1*, *ZmSSI*, *ZmSSIIa,* and *ZmSSIIIa* were increased, while those of *ZmSh2* and *ZmSSIV* reduced significantly in the endosperm of the transgenic maize. Overall, this study shows that ZmTCP7 functions as a transcriptional repressor of *ZmBt2* and a negative regulator of endosperm starch accumulation, providing new insights into the regulatory networks that govern *ZmBt2* expression and starch biosynthesis pathway in maize.

## Introduction

The process of starch biosynthesis is a major developmental event that determines the final grain yield and quality in maize (*Zea mays* L.) and in other cereals (Olsen and Becraft, 2013; Olsen, 2020). The global economic importance of maize is unmatched by any other commodity, as it provides more than 80% of the calories in the human diet and livestock feed, and also serves as primary products for a variety of food and non-food industries, due to its high starch content (Preiss, 2018; Qu et al., 2019; Olsen, 2020). Maize starch, which is one of the highest quality starches worldwide, contributes ~91% of the estimated global production of cereal starches and accounts for about 70% of the final weight of the mature kernels (Agama-Acevedo et al., 2019; Qu et al., 2019). This possibly explains why maize has been extensively used as a model crop in the study of the genetic and molecular basis of starch biosynthesis in cereals.

Starch biosynthesis in cereal crops involves a series of highly coordinated networks of biochemical reactions through the actions of a suite of key metabolic enzymes. The first committed step begins with the formation of adenosine diphosphate glucose (ADPG) from glucose-1-phosphate (G1P) and adenosine triphosphate (ATP) by the regulatory action of the adenosine diphosphate-glucose pyrophosphorylase (AGPase; Hannah, 2007). In cereals, ADPG is mainly synthesized in the cytosols, and it is simultaneously imported into and strictly committed to starch formation within the amyloplast (Denyer et al., 1996; James et al., 2003; Hannah, 2007; Cossegal et al., 2008). The imported ADPG is then used as a substrate by granule-bond starch synthase 1 (GBSS1) and soluble starch synthase (SS) isozymes to prime and elongate the α-1, 4-linked glucan chains of amylose and amylopectin, respectively. Subsequently, branch links are introduced into amylopectin growing chains at α-1, 6 by starch branching enzymes (SBEs), and improperly placed branches are specifically trimmed by starch debranching enzymes (DBEs; Hannah, 2007; Tetlow and Emes, 2017; Preiss, 2018). The various isoforms of these key starch-associated enzymes are encoded by uniquely different genes, and their functions have been clarified in great detail using loss and gain of function genetic approaches (Hannah, 2007; Cossegal et al., 2008; López-González et al., 2019; Olsen, 2020).

Among the key starch metabolic enzymes, AGPase attracts great attention from cereal researchers. Due to its catalytic control of the first committed step, AGPase exerts substantial control over ADPG, the glucose donor required for starch formation, and directly determines the level of seed endosperm starch and the final grain yield (James et al., 2003; Hannah, 2007; Saripalli and Gupta, 2015; Tetlow and Emes, 2017). AGPase functions as heterotetramers and is composed of two identical small and two identical large subunits, each of which is encoded by different genes that differentially evolved with distinct functions (Preiss et al., 1990; Yan et al., 2009). In maize, the endosperm-specific genes *brittle2* (*ZmBt2*) and *shrunken2* (*ZmSh2*) encode small and large AGPase subunits, respectively (Cossegal et al., 2008; Saripalli and Gupta, 2015; Hannah et al., 2017).

Since its identification, *ZmBt2* has continued to be a gene of interest due to its role in the rate-limiting step of the starch biosynthesis pathway (Hannah and Nelson, 1976; Bae et al., 1990; Preiss et al., 1990; Cossegal et al., 2008; Geigenberger, 2011; Hannah et al., 2017; Zhang et al., 2019). Lack or inactivation of *ZmBt2* has been associated with low AGPase activity, reduced accumulation of endosperm starch, shrunken/collapsed kernel phenotype, and significant modification of the carbohydrate metabolic pathway in seed endosperm (Hannah and Nelson, 1976; Bae et al., 1990; Preiss et al., 1990; Cossegal et al., 2008; Zhang et al., 2019). Knowledge of the molecular function of *ZmBt2* has offered a better understanding of the metabolic networks of *ZmBt2* in maize. However, regulation of *ZmBt2* at the transcriptional level remain elusive. Transcriptional regulation plays a key role in modulating the expressions of genes related to starch biosynthesis, and growing evidence shows that *ZmBt2* expression is temporally and spatially governed by transcriptional regulators (Cossegal et al., 2008; Geigenberger, 2011; Li et al., 2018). A transcription factor (TF) activates or inhibits the transcription of a gene by targeting *cis*-acting regulatory elements on the gene promoter. This ensures that the correct gene is expressed (Stower, 2011).

An earlier study in our lab identifies a short functional fragment of *ZmBt2* promoter [~394-bp; from −370 upstream of the transcription start site (TSS) to +24] and further shows that it contains multiple putative *cis*-acting regulatory elements (Li et al., 2018), indicating that several TFs may be controlling *ZmBt2* expression. Differential expression of *ZmBt2* has been shown to produce AGPase with variable sensitivity with considerable changes in endosperm starch content (Cossegal et al., 2008; Geigenberger, 2011; Zhang et al., 2019). Thus, transcriptional regulation of *ZmBt2* is critical for starch accumulation level in maize endosperm. Currently, only two functionally redundant TFs, ZmNAC128 and ZmNAC130, have been identified as *ZmBt2* regulators controlling starch accumulation in maize endosperm (Zhang et al., 2019). To improve grain yield and quality of maize via planned modification of endosperm starch content, it is necessary to identify and characterize all transcription factors that affect *ZmBt2* expression.

Generally, little progress has been made in the area of transcriptional regulation of starch biosynthesis in maize, as only a few reports are currently available. For example, some endosperm-specific TFs, including O2, PBF, ZmDOF36, ZmEREB156, ZmbZIP91, and ZmNAC34, have been reported to be involved in the regulation of starch biosynthesis by targeting and governing the expressions of different genes related to starch biosynthesis (Chen et al., 2016; Huang et al., 2016; Zhang et al., 2016; Peng et al., 2019; Wu et al., 2019). To allow for a predictable improvement in grain yield and quality, more information on the transcriptional control of genes involved in starch biosynthesis in maize is needed.

The TCP proteins, named after the first known members (TB1, CYC, and PCFs), are phylogenetically related plant-specific TFs that share a highly conserved TCP domain, which harbors a non-canonical basic-helix–loop–helix (bHLH) structural motif. The TCP domain is involved in DNA binding and protein dimerization (Cubas et al., 1999; Kosugi and Ohashi, 2002). TCP TFs are classified into two divergent groups; TCP class I and TCP class II, and the later is further divided into two clades; CIN and CYC/TB1 (Cubas et al., 1999; Martín-Trillo and Cubas, 2010). TCP TFs are functionally diversified throughout the plant kingdom and have been characterized as regulators of multiple growth and development pathways, and their activities determine plant form and architecture (Cubas et al., 1999; Danisman et al., 2012; Manassero et al., 2013; González-Grandío and Cubas, 2016; Feng et al., 2018; Bao et al., 2019; Perez et al., 2019). Currently available evidence has shown that most TCP class I genes are activators of plant development, whereas TCP class II members often function as a repressor of various growth and development pathways (González-Grandío and Cubas, 2016; Lucero et al., 2017; Sarvepalli and Nath, 2018).

In this study, we used a short functional fragment of *ZmBt2* promoter (394 bp) that was characterized in our previous work (Li et al., 2018), to screen a library of protein cDNAs from maize endosperm in a yeast one hybrid (Y1H) screening system. We identified a novel endosperm preferentially expressed transcription factor, ZmTCP7, which suppressed *ZmBt2* transcription and consequently reduced the activity of the AGPase enzyme and starch content in the endosperm of transgenic maize overexpressing *ZmTCP7*. We showed that ZmTCP7 is a transcriptional repressor of *ZmBt2* gene and a negative regulator of starch biosynthesis pathway in maize endosperm. Therefore, this study provides new information on understanding the regulatory networks that govern *ZmBt2* expression and the starch biosynthesis pathway in maize.

## Materials and methods

### Plant materials

The maize B73 inbred line was grown at the experimental field of Sichuan Agricultural University, Chengdu, China, under recommended agronomic guidelines and was self-pollinated. Developing kernels between 3 and 25 days after pollination (DAP), leaves, roots, stems, and flowers were sampled at 10 DAP and immediately frozen in liquid nitrogen and stored at *−*80°C until use. Each tissue sample was obtained from at least three plants.

The maize B104 inbred line was used to generate the overexpression lines (OE-1 and OE-2) used in this study. Transgenic lines and wild-type (WT) were grown at the research stations of Sichuan Agricultural University in Wenjiang (Sichuan province) and Sanya (Hainan province), China, under recommended agronomic conditions. For each transgenic line, developing endosperms at 16 and 20 DAP and mature ears were harvested from at least three plants. The developing endosperms were removed from the seed coat and embryo, and promptly frozen in liquid nitrogen and stored at −80°C pending processing.

### Construction of the cDNA library and cloning of the *ZmBt2* promoter

The maize endosperm cDNA prey library was constructed by OEBiotech Company, Shanghai. The pGADT7-Rec vector containing the GAL4-AD system was used to construct the library of protein cDNAs synthesized from mRNA pooled from endosperms from the maize inbred line B73 at different stages of development.

We amplified a short functional region of the *ZmBt2* promoter [identified in our previous study (Li et al., 2018)] from −370 upstream of the transcription start site (TSS) to +24 (394 bp) and named it p*Bt2*P1. Subsequently, we inserted the amplification product into the pMD19-T vector, and subcloned into the pAbAi vector upstream of the AbAr reporter gene (AUR1-C) at the H*ind*III and X*ho*I sites to generate pAbAi-*Bt2*P1 as bait vector. Activation of AbAr, the reporter gene harbored by the pAbAi vector, confers screens with resistance to the Aureobasidin A (AbA) antibiotic in the Y1H screening system. All primers are listed in Supplementary Table S1.

### Yeast one hybrid library screening

The Yeast one hybrid (Y1H) library screening system was used to screen for candidate TF(s) that putatively target and interact with p*Bt2*P1, following the protocols described in the User’s Manual of the Matchmaker^®^ Gold Y1H Library Screening System (Clontech, United States). The Y1HGold yeast strain was used in this study. In short, the pAbAi-*Bt2*P1 bait construct was linearized by B*st*BI and integrated into the genome of Y1HGold yeast strain by homologous recombination to generate the bait-specific reporter strains Y1HGold (*Bt2*P1/AbAi) and determined 150 ng/ml AbA as minimal concentration that completely suppresses colony growth of the reporter strains. Then, cDNA prey library AD-vector was transformed into the Y1HGold (*Bt2*P1/AbAi) reporter strains. The yeast suspension aliquots were diluted to 10^−1^, 10^−2^, and 10^−3^, and 100 μl of each dilutant was spread in SD/-Leu plates to evaluate the total number of colonies screened. Furthermore, Y1HGold (*Bt2*P1/AbAi)/pGADT7-Rec and Y1HGold (p53/AbAi)/pGADT7-Rec/p53 yeast transformants were generated as negative and positive control systems, respectively. All yeast transformants were then spread in SD/-Leu/^+^AbA^150 ng/ml^ and incubated at 28°C for 3–4 days. Healthy single clones responsible for reporter activation were selected from SD/-Leu/^+^AbA^150 ng/ml^ plate, and were transferred to fresh SD/-Leu/^+^AbA^150 ng/ml^, SD/-Leu/^+^AbA^175 ng/ml^ and SD/-Leu/^+^AbA^200 ng/ml^ media plates, and then, incubated at 28°C for 3–4 days to confirm genuine interactions. Library plasmids were transformed into *E. coli*, DH5α strain, isolated and purified following colony PCR and were sequenced with T7 sequencing primer at Sangon Genomics Institute (Shanghai, China). The sequencing results were analyzed against maize genome using the BLAST tool in Ensembl Plants database,^1^ and ZmTCP7 belonging to the TCP TF family was identified as candidate TF.

### Confirmation of the Y1H interaction in an individual assay

The complete ZmTCP7 encoding sequence was first cloned into the pMD19-T vector, and then, subcloned into pGADT7-Rec vector at N*de*I and B*am*HI sites to produce pGADT7-Rec/ZmTCP7 as a specific prey construct. The construct was transformed into bait-reporter strains of Y1HGold (ZmBt2P1/AbAi) as previously described. The transformants along with the controls were spread in SD/-Leu/^+^AbA^150 ng/ml^, SD/-Leu/^+^AbA^175 ng/ml^, and SD/-Leu/^+^AbA^200 ng/ml^ plates, and incubated at 28°C for 3 days to validate the activation of the bait reporter by ZmTCP7. All primers are listed in Supplementary Table S1.

### Annotation of the protein domain and phylogenetic analysis

For diagnostic domain analysis, the protein sequence of ZmTCP7 was submitted to the Interpro database (Blum et al., 2021) and searched against the Interpro’s signatures using default settings. To obtain homologs of ZmTCP7 in other crops, the ZmTCP7 protein sequence was used as a query to perform a BLASTp search using the UniProt BLAST tool (UniProt Consortium, 2021). Multiple alignment of the protein sequences of the identified homologs was performed using MEGA X, and the phylogenetic tree was constructed using the neighbor joining method with 1,000 bootstrap replicas (Saitou and Nei, 1987; Kumar et al., 2018). Evolutionary distances were computed using the JTT matrix-based method (Jones et al., 1992).

### Analysis of ZmTCP7 transactivation

The transactivation activity of ZmTCP7 was investigated using the yeast expression plasmid, pGBKT7, based on the yeast GAL4 system. The full length of the coding sequence of ZmTCP7 was fused at the E*coR*I and B*amH*I sites in frame with the GAL4 binding domain in the pGBKT7 plasmid. The pGBKT7-ZmEREB94 previously reported to show strong transactivation activity (Li et al., 2017) was used as a positive control, and pGBKT7 as negative control. All constructs were transformed into the yeast strain AH109 according to the procedures described in the Yeastmaker^™^ Yeast Transformation System (Clontech, PT1172-1). The yeast transformants were selected on SD/-Trp and transactivation activity was evaluated on SD/-Trp-His-Ura-Ade/^+^X-α-gal plates. The yeast cells on the selection and screening plates were incubated at 28°C for 3–4 days.

### Subcellular location of ZmTCP7

The expression vector, pCAMBIA2300-35S-eGFP (Xiao et al., 2017), containing an enhanced green fluorescent protein (eGFP) under the control of CaMV 35S promoter, was used for the analysis of subcellular localization of ZmTCP7 in maize endosperm protoplast system (MEPS; Hu et al., 2020). The ZmTCP7 coding sequence without stop codon was amplified and inserted into the expression vector at the K*pn*I and X*ba*I sites to create the fusion construct pCAMBIA2300-35S-ZmTCP7-eGFP. Maize endosperm protoplast isolation and transfection were carried out as described by Hu et al. (2020). About 100 μl of protoplast cells at density 2 × 10^6^ cells/ml were transfected with 10 μg plasmid DNA from the fusion construct and incubated at 28°C for 16 h. Before fluorescence microscopy, transfected protoplast cells were incubated with 0.1 μg mL^−1^ 4,6-diaminodino-2-phynylindole (DAPI) for 5 min. The intracellular GFP and DAPI signals of the protoplast cells were observed with a confocal microscope using the Leica Application Suite X (Leica Microsystems, Germany).

### Transient overexpression analyses in maize endosperm protoplast system

The maize endosperm protoplast system (MEPS) was used to transiently investigate the regulatory influence of ZmTCP7 on the *ZmBt2* expression. The open reading frame of ZmTCP7 along with the *BamH*I and *Sac*I restriction sites was amplified and cloned into pMD19-T. The resulting construct was digested and subcloned in the pUbi-Gus vector (Hu et al., 2011) at the *BamH*I and *Sac*I sites to produce pUbi-ZmTCP7-Gus as an effector expression plasmid, under the control of the maize *ubiquitin* promoter. pUbi-ZmTCP7-Gus was used for transient overexpression in the MEPS. About 100 μl of protoplast cells was transfected with 20 μg plasmid DNA from the pUbi-ZmTCP7-Gus construct. A separate transfection was also performed with empty pUbi-Gus vector and used as control system (WT). The transfected cells were incubated at 28°C for 24 h and total RNA was isolated from cells and used for expression analysis.

To identify the region that contains the binding site targeted by ZmTCP7, the 394-bp sequence of p*Bt2*P1 and its four 5′truncate derivatives including deletions of 294, 244, 210, and 67-bp were amplified from pAbAi-*Bt2*P1 with five forward primers and one common reverse. Subsequently, the product of each amplification with *Pst*I and *BamH*I restriction sites was inserted into pMD19-T (Takara) and, thereafter, digested and purified. The digested fragments were used to replace the full length of zaS3 in the plasmid pzaS3-ALuc (Hu et al., 2011) at the *Pst*I and *BamH*I sites to generate pBt2P1-ALuc, pBt2P2-ALuc, pBt2P3-ALuc, pBt2P1.1-ALuc, and pBt2P1.2-ALuc, as expression reporter constructs. All primers are listed in Supplementary Table S1. The construct pUbi-ZmTCP7-Gus and the vector pUbi-Gus were used as an effector and an internal control, respectively. About 8 μg plasmid DNA each from the reporter and effector constructs and 4 μg of the internal control (pUbi-Gus) were cotransfected into 100 μl of protoplast cells and incubated at 28°C for 24 h as described in Hu et al. (2020). The fluorogenic assay for β-glucuronidase activity (Gus) was determined using the method described by Hu et al. (2011), and the activity of luciferase (Luc) was analyzed using the Luciferase Reporter^®^ Assay System (Promega, United States), and both were determined on a Luminoskan^™^ Ascent Luminometer (Thermo Scientific). At least five independent experiments and three technical replicates were performed for each experiment. Subsequently, the putative *cis*-acting region from −270 and −220 bp (50 bp) determined from p*Bt2*P1 dissection was searched for previously published consensus motifs, GGNCCCAC and GTGGNCCC for TCP classes I and II, respectively (Kosugi and Ohashi, 2002), to identify the candidate binding site targeted by ZmTCP7.

### Electrophoretic mobility shift assay

The full length of the ZmTCP7 coding sequence was amplified and cloned in a frame with the GST tag at the *EcoR*I and *Xho*I sites of the expression vector, pGEX-6-1, to generate the GST-ZmTCP7 fusion protein construct. The construct was transformed into *Escherichia coli* BL21 (DE3) cells, grown at 37°C until the optical density at 600 nm was 0.6 and then, cooled down for 15 min at 4°C. Afterwards, the expressed fusion protein was induced by adding isopropyl β-D-1-thiogalactopyranoside (IPTG) to the cultured solution at a final concentration of 0.25 mM, and incubated at 16°C, 150 rpm for 12 h. The GST-ZmTCP7 fusion-protein was purified using GST-tagged fusion protein purification kit (Thermo Scientific), and stored in aliquots at -80°C pending usage.

Biotin labeled oligonucleotide probes of the wild-type (WT: containing genuine binding site) and its mutant (Mu: containing mutated binding site) were synthesized by Sangon Biotech (Shanghai, China). The 3′end biotinylated double-stranded oligonucleotides of each pair were annealed in ultrapure water at 95°C, 5 min; 100 cycles 95°C (−0.1C/cycle), 1 min; 4°C hold in a thermocycler. An unlabeled oligonucleotide probe was also synthesized and used for the competitor experiment. The biotin labeled and non-labelled oligonucleotide probes were incubated with aliquot of purified GST-ZmTCP7 fusion-protein in electrophoretic mobility shift assay (EMSA) buffer at 25°C for 30 min, electrophoresed by 8% polyacrylamide gel (PAGE), cross-linked, following the procedures provided in LightShift^®^ Chemiluminescent EMSA kit (Thermo Scientific) and, visualized on Chemiluminescent imaging system (Thermo Scientific).

### Vector construction and maize transformation

To develop transgenic maize lines overexpressing *ZmTCP7*, we first replaced the CaMV 35S promoter that drives the expression of Bar gene selectable marker in pCAMBIA3301 vector with *ubiquitin* promoter to generate pUB3301-eGus. Subsequently, we amplified the entire fragment of the *ubiquitin* promoter along with the ZmTCP7 coding sequence (Ubi-ZmTCP7) from the expression plasmid pUbi-ZmTCP7-Gus and subcloned it into the overexpression vector pUB3301-eGus at H*ind*III and P*mlI* to generate the overexpression construct pUB3301-eGus-ZmTCP7. The transcription of ZmTCP7 is operationally driven by the maize *ubiquitin* promoter and ended by NOS terminator sequence of the vector. The Bar gene of the construct was used in screening for transgenic-positive seedlings and kernels. The elite maize B104 inbred line was used in the development of transgenic lines. To generate maize transgenic plants, the overexpression construct: pUB3301-eGus-ZmTCP7, was integrated into healthy immature embryos derived from developing kernels at 10 DAP using the *Agrobacterium tumefaciens-*mediated transformation system described in other studies (Ishida et al., 2007; Zhong et al., 2018). We obtained two independent T_0_ transgenic lines (OE-1 and OE-2). The plant leaves at stage V6 were used for the bar strip test using the GeneTrue^™^ BAR Test Kit (Artron, Canada) to detect transgenic positive plants. The plants of the two T_0_ transgenic lines were grown at the research station of Sichuan Agricultural University in Wenjiang and backcrossed to the maize inbred B104 to generate T_0_ seeds. Kernels of developing ear at 16 DAP were obtained for expression analysis. T_0_ seeds were planted in Sanya (Hainan province) and managed under recommended agronomic conditions to produce T_1_ plants and seeds and subsequently advanced to T_2_. Positive transgenic plants were identified using Bar strip test and were cross-bred with the WT. Kernels of the developing ear at 20 DAP were obtained for the enzyme activity assay. The bar strip test was performed with the GeneTrue^™^ BAR Test Kit (Artron, Canada) to detect transgenic-positive kernels. The non-transformed kernels were used as WT in the different gene expression analyses. Some transgenic positive plants were self-pollinated, and developing endosperm at 20 DAP and mature kernels obtained from the ears of at least 10 individual T_2_ plants were then used in the analyses of enzyme activity and starch content, respectively.

### AGPase activity assay

AGPase activity was determined *in vitro* using developing endosperm at 20 DAP. Ten kernels each of OE-2 and WT were selected and their embryo and pericarp removed leaving the endosperms. The endosperms were ground in liquid nitrogen and ~100 mg was promptly transferred into 1.5ml microcentrifuge tube containing 1 ml extraction buffer (50 mM Tris–HCl, pH 8.0, 10 mM EDTA-Na_2_.2H_2_O, 5 mM DTT, 1 mM PMSF, and 10% (v/v) glycerol) on ice. The suspension was mixed in a vortex for 3 s and centrifuged at 13, 000 ×g for 20 min, at 4°C. The supernatant was quickly transferred to a new microcentrifuge tube and kept on ice to be used for the AGPase activity assay. The crude protein concentration was determined using a BCA protein assay kit (BOSTER, AR0146), following the user’s manual. AGPase activity assay performed with a 5 μl undiluted crude extract in a total of 100 μl standard reaction containing 50 mM Hepes, 3.67 Mm ADPG, 0.1 M PPi, 0.1 M MgCl_2_ and 0.1 M DTT. The AGPase activity was determined using the enhanced ATP assay kit (Beyotime) following the user manual. Three technical replications were performed.

### Total starch determination

About 10 g of mature T_2_ kernels from ears of the two transgenic overexpression lines and WT were milled to powder. Total starch content was determined following the instructional manual in the Total Starch Assay kit (K-TSTA-50A, Megazyme), based on the thermostable amyloglucosidase (AMG) and α-amylase method. About 100 mg of the kernel powder was weighed in a corning culture tube and 0.2 ml of aqueous ethanol (80% v/v) was added and vortexed to aid in dispersion. The mixture was resuspended with 2 ml of cold sodium hydroxide (1.7 M) and vortexed for 15 s and stirred with a magnetic stirrer in an ice water bath for 15 min with intermittent mixing in a vortex at a 5-min interval. Then, 8 ml sodium acetate buffer (600 mM, pH 3.8) was added and vortexed. Instantly, 0.1 ml of undiluted thermostable α-amylase and 0.1 ml of AMG (3,300 U/ml) were added and mixed in a vortex for 3 s. The entire mixture was incubated in a 50°C water bath for 30 min and then cooled to room temperature. Then 2 ml of the solution was taken and transferred to a microfuge tube and centrifuged at 13,000 ×g for 5 min. Then, 1.0 ml aliquot of the supernatant was transferred into 15 ml tube containing 4 ml sodium acetate buffer (100 mM, pH 5.0) and mixed well. A 0.1 ml duplicate aliquot was then carefully transferred to the bottom of a glass test tube. Then, 3.0 ml of GOPOD reagent was added and incubated at 50°C for 20 min, and the absorbance against the blank reagent was measured at 510 nm.

### RNA isolation and RT-qPCR

Total RNA was isolated from 100 mg of frozen developing endosperm and other tissues stored at −80°C, and protoplast cells using TRIzol reagent (Invitrogen), treated with DNase I and purified with Total RNA Extraction Kit (Tianmo Biotech, Beijing). cDNA was synthesized from 1 μg total RNA using the PrimeScript RT reagent kit (Takara). qPCR was performed with the SYBR Green PCR Master Mix (Takara) on the CFX Real-Time PCR System (Bio-Rad, United States). Each PCR reaction was performed at least with three technical replicates. The relative gene expression was analyzed by using 2^−∆∆Ct^ with the maize *Actin 1* gene (GRMZM2G126010) as the internal reference gene. All primers used for qPCR are listed in Supplementary Table S1.

## Results

### Identification of the ZmTCP7 transcription factor

The endosperm-specific *brittle* 2 gene, *ZmBt2*, which encodes the small subunit of AGPase enzyme, has long been characterized to play important role in maize endosperm starch biosynthesis (Bae et al., 1990; Preiss et al., 1990; Cossegal et al., 2008). However, the mechanism of its transcriptional regulation is not well understood. To understand the modulation of starch biosynthesis in maize endosperm through transcriptional regulation of *ZmBt2* expression, we used a minimal fragment of the *ZmBt2* promoter from −370 to +24 (394 bp; p*Bt2*P1), characterized in our previous study as the regulatory region of the promoter (Li et al., 2018), to screen a library of protein cDNAs from maize endosperm in a Y1H screening system.

**Figure 1:**
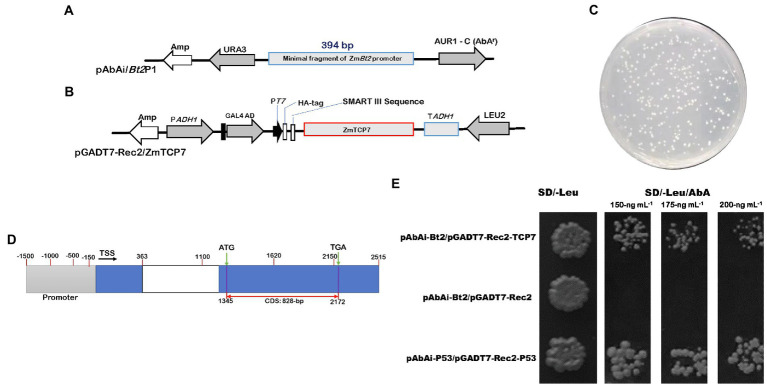
Detection of positive transformants and specific interaction between ZmTCP7 and the *ZmBt2* promoter in a yeast-one hybrid system. **(A,B)** Schematic diagrams of the bait (pAbAi/*Bt2*P1) and prey protein (pGADT7-Rec2/ZmTCP7) constructs, respectively. AUR1-C/AbA^r^ is an antibiotic reporter gene that confers resistance to the Aureobasidin A (AbA) antibiotic. AbAr expression is driven by positive DNA-protein interactions. URA3 and Leu2 are yeast selectable nutritional markers for yeast replication and selection. The bait (pAbAi/*Bt2*P1) contains the fragment of *ZmBt2* promoter (p*Bt2*P1; 394-bp) fused upstream of the AbA^r^ reporter gene, while the construct of the prey protein (pGADT7-Rec2/ZmTCP7) harbors GAL4-AD/ZmTCP7 fusion protein; **(C)** 100^−2^ dilution of library transformants screened in SD/-Leu selective medium supplemented with an AbA antibiotic of 150 ng ml^−1^ concentration; **(D)** Schematic diagrams of the ZmTCP7 genome model showing the promoter region (gray rectangle), exons (blue rectangle), intron (white rectangle), transcription start site (TSS), and the complete coding sequence (CDS); **(E)** 100^−2^ dilution of yeast transformants screened on two selective medium: (i) SD/-Leu and (ii) SD/-Leu supplemented with different concentrations of AbA antibiotic: 150 ng ml^−1^, 175 ng ml^−1^, and 200 ng ml^−1^. The transformants harboring pAbAi-*Bt2*P1/pGADT7-Rec and pAbAi-P53/pGADT7-Rec-P53 were used as negative and positive control systems, respectively.

Initially, we generated the plasmid pAbAi-*Bt2*P1 (Figure 1A), which harbors the minimal *ZmBt2* promoter fragment upstream of the AbAr reporter gene that confers resistance to the AbA antibiotic, as a bait-specific reporter construct. Generally, the expression of AbAr is driven by positive interactions between the bait DNA sequence and potential prey proteins. Following the construction of the bait reporter plasmid (pAbAi-*Bt2*P1), we transformed the yeast strain Y1HGold with the pAbAi-*Bt2*P1 to create bait-specific reporter yeast strain [Y1HGold (*Bt2*P1/AbAi)]. We then tested the background expression of AbAr (antibiotic resistance gene) to determine the minimum concentration of AbA antibiotics that can adequately suppress endogenous activation of the Y1HGold (*Bt2*P1/AbAi) reporter yeast strain. The AbA concentration of 150 ng ml^−1^ was found to be minimally sufficient to inhibit endogenous activation of the Y1HGold (*Bt2*P1/AbAi) reporter yeast strain. The 150 ng ml^−1^ AbA concentration was used to supplement SD/-Leu selective medium in the screening of the Y1HGold (*Bt2*P1/AbAi) reporter yeast strain transformed with the library construct of the prey protein cDNAs (Figure 1C). After initial screening, a total of 283 colonies were obtained from yeast cell suspension of 10^−2^ dilutant spread on SD/-Leu plate, resulting in ~4.28 × 10^6^ colonies that were screened. For identification analysis, 130 large healthy colonies that activate the reporter were selected from SD/-Leu/AbA^150 ng/ml^ plate and rescreened in fresh selective medium supplemented with 150 ng ml^−1^ and higher concentrations (175 ng ml^−1^ and 200 ng ml^−1^) of AbA, to confirm genuine interactions. Subsequently, we performed colony PCR to eliminate duplicates or abundant interactors, and consequently selected 50 PCR products for sequencing and were analyzed using a BLASTx search against the maize genome in the Ensembl Plants database. Interestingly, we isolated multiple independent cDNAs that encode an uncharacterized transcription factor (GRMZM2G035944) annotated as ZmTCP7 in the Maize genetics and genomic database, MaizeGDB.^2^ Although the BLASTx search identified several functional and uncharacterized proteins, ZmTCP7 was the only TF (regulatory protein) obtained from the sequenced clones and, therefore, was used as the candidate TF in this study.

To confirm that the ZmTCP7 TF interacts with the functional fragment of *ZmBt2* promoter, we constructed prey-specific-AD plasmid (pGADT7-Rec-*ZmTCP7*: Figure 1B) with the ZmTCP7 predicted coding sequence (828 bp: Figure 1D) and transformed into the bait-specific reporter yeast strain of Y1HGold (*Bt2*P1/AbAi). Then, the transformants were screened in the SD/-Leu selective medium supplemented with 150 ng ml^−1^, 175 ng ml^−1^, and 200 ng ml^−1^AbA. The growth of yeast colonies in the selective medium validated the activation of the reporter by ZmTCP7 TF in the Y1H assay (Figure 1E), suggesting that ZmTCP7 can directly bind to the *ZmBt2* promoter and regulate its activity through its interaction. The role of ZmTCP7 TF in the transcriptional regulation of *ZmBt2* expression and starch biosynthesis in maize was further investigated.

### ZmTCP7 is an endosperm preferentially expressed transcription factor

**Figure 2:**
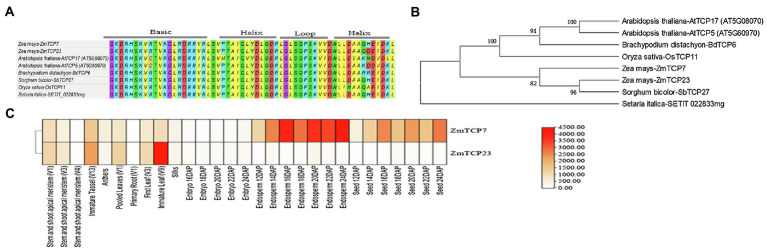
Domain signature, phylogenetic relationships of ZmTCP7 and its homologs, and expression heat map of *ZmTCP7* and its paralog (*ZmTCP23*) in maize. **(A)** Alignment of the TCP domain of ZmTCP7 and its homologs in maize, rice, foxtail millet, sorghum, *Arabidopsis* and *Brachypodium*; **(B)** Phylogenetic tree of ZmTCP7 and its homologs in maize, rice, foxtail millet, sorghum, *Arabidopsis* and *Brachypodium*. Multiple alignment of the protein sequences of ZmTCP7 and its homologs was performed using MEGA X, and the phylogenetic tree was constructed using the neighbor joining method with 1,000 bootstrap replicas (Saitou and Nei, 1987; Kumar et al., 2018). Evolutionary distances were calculated using the JTT matrix-based method (Jones et al., 1992); **(C)** Heat map showing the expression of *ZmTCP7* and its paralog (*ZmTCP23*) in different maize tissues. High, medium and low expression levels are indicated in red, orange and white, respectively.

ZmTCP7 was predicted to encode 275 amino acid residues. We subjected the ZmTCP7 protein sequence to domain annotation using the Interpro domain database (Blum et al., 2021) and found that ZmTCP7 contains a TCP domain, which harbors a noncanonical bHLH structural motif (Figure 2A), at amino acids 57–118.

Subsequently, we obtained TCP TF protein sequences in maize, rice, and *Arabidopsis* from online resources^3^^,^^4^ and used them in phylogenetic analysis. Our result indicated that ZmTCP7 belongs to the *CIN* clade of class II TCP (Supplementary Figure S1), which is consistent with previous reports on the evolutionary and phylogenetic relationships of TCP proteins in maize (Chai et al., 2017; Ding et al., 2019). Following this, we constructed a phylogenetic tree with the protein sequences of ZmTCP7 and its homologs in maize, rice (*Oryza sativa*), *Arabidopsis thaliana*, *Brachypodium distachyon*, sorghum (*sorghum bicolor*), and foxtail millet (*Setaria italica*). Phylogenetic relationships and bootstrap values indicated that ZmTCP7 is highly conserved in maize and other crop species (Figure 2B). Currently, none of these homologs has been functionally characterized. The ZmTCP7 homolog in sorghum (SbTCP27) showed closer relationship to maize than those of other monocots, and paired with ZmTCP23 in maize (Figure 2B). A previous evolutionary study revealed that both ZmTCP7 and ZmTCP23 were a pair of paralogous genes, duplicated about 7.75 million years ago through a segmental duplication event (Chai et al., 2017).

Sequence alignment analysis showed that ZmTCP23 shared only 78.3% identity with ZmTCP7 (Supplementary Figure S2). To have an important clue about the molecular route of regulatory action of ZmTCP23 and ZmTCP7, we obtained their gene expression data of different tissues at specific developmental stages, from the maize gene expression atlas available at MaizeGDB,^5^ since sequence similarity is not always sufficient to determine and infer gene function (Chai et al., 2017). Thereafter, we constructed a heatmap with the expression data to investigate their expression profiling. As shown by the heat map (Figure 2C), both genes were expressed in all tissues, but their expression patterns were different in the various tissues. Specifically, ZmTCP7 was predominantly expressed in endosperms and seeds, while ZmTCP23 was mainly expressed in leaves and tassel (Figure 2C), suggesting that both genes may not have the same regulatory function. Divergence of expression patterns in paralogous gene pairs may lead to evolutionary diversification of gene function (Chai et al., 2017). Thus, ZmTCP7 and ZmTCP23 may have diverged for evolution of novel gene function or regulatory network in maize, but their specific function still remains to be determined.

**Figure 3:**
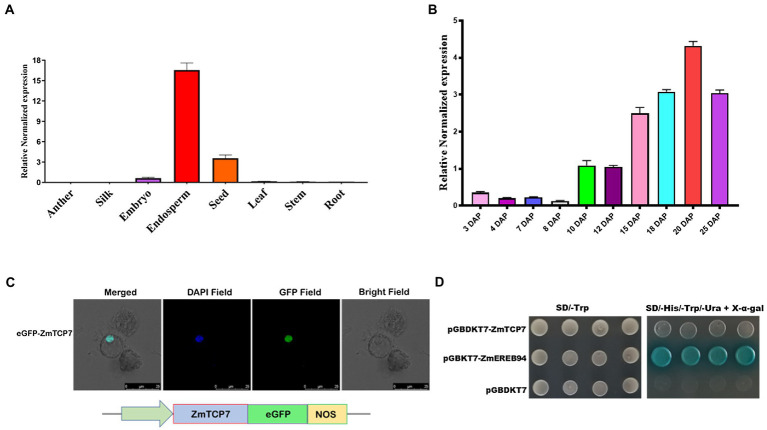
Spatiotemporal expression pattern and transcription factor characteristics of ZmTCP7. **(A)** Tissue-specific expression analysis of *ZmTCP7* in various maize tissues by RT-qPCR. Developing endosperm and seed at 16 DAP and other tissues at 10 DAP from at least three plants were used for the tissue-specific expression analysis; **(B)** analysis of expression of *ZmTCP7* in developing kernels between 3 and 25 DAP by RT-qPCR. The bars indicate the mean ± SD of three replicates; **(C)** ZmTCP7-eGFP fusion construct (lower panel) and subcellular location of the ZmTCP7-eGFP fusion protein in maize endosperm protoplast cells (upper panel): GFP, green fluorescent signal, DAPI (4,6-diaminodino-2-phynylindole), nuclear marker. Scale bar = 25 μm; **(D)** analysis of ZmTCP7 transactivation in the yeast system. pGBKT7-ZmEREB94 and pGBKT7 are positive and negative controls, respectively.

We then performed temporal and spatial expression patterns by reverse transcriptase quantitative PCR (RT-qPCR) in 8 maize tissues: anther, silk, leaf, stem, root, seed, endosperm, and embryo, to confirm tissue-specific expression profiles of ZmTCP7. The RT-qPCR results confirmed that *ZmTCP7* transcript was predominantly expressed in maize endosperm (Figure 3A), with its transcript relatively maintained at low-level during early development (3–8 DAP), and gradually accumulates and maintains a high level from 10 to 25 DAP (Figure 3B) during grain filling. Furthermore, the *ZmTCP7* transcript was also present moderately in the seed, fairly expressed in the embryo, but was weakly expressed in the anther, silk, leaf root, and stem (Figure 3A). It is evident that the endosperm, seed, and embryo are the major organs mostly expressing ZmTCP7 relative to other tissues. However, embryo is not strongly expressing ZmTCP7 compared with the endosperm and seed. Therefore, ZmTCP7 may plays an important regulatory role in endosperm and seed development in maize.

In order to examine the subcellular localization of ZmTCP7, we generated a pCAMBIA2300-35S-ZmTCP7-eGFP fusion construct (Figure 3C; lower panel), by inserting its coding sequence without the termination codon in frame with the eGFP reporter driven by CaMV35S promoter, and transiently expressed the recombinant vector in maize protoplast cells. The transfected protoplasts were then stained and incubated with DAPI, a nuclear staining dye, and subsequently observed under a laser confocal microscope. The overlap of the florescent signals of the GFP and the nuclear marker in the nucleus (Figure 3C; upper panel) clearly indicated that ZmTCP7 was targeted and can function in the nucleus.

Being a nuclear protein and TF, we examined the autonomous transcriptional activity of ZmTCP7 in yeast system. We fused the entire length of the ZmTCP7 coding sequence in frame with the GAL4-BD of the pGBKT7 plasmid and, thereafter, transformed the obtained construct (*pGBKT7-ZmTCP7*) into the yeast strain AH109. Also, pGBKT7-ZmEREB94, which has been reported to have strong transactivation activity (Li et al., 2017), and empty pGBKT7 were transformed into the AH109 yeast strain and used as positive and negative controls, respectively. The yeast transformants harboring *pGBKT7-ZmTCP7,* just as the negative control, could not reveal visible β-galactosidase activity in the SD/-Trp-His-Ura-Ade/^+^X-α-gal medium in contrast to the positive control, which displayed strong autonomous transcriptional activity (Figure 3D). These results suggest that ZmTCP7 does not exhibit transactivation activity in yeast system.

### ZmTCP7 targets GAACCCCAC site in the promoter of *ZmBt2* gene to suppress its expression

**Figure 4:**
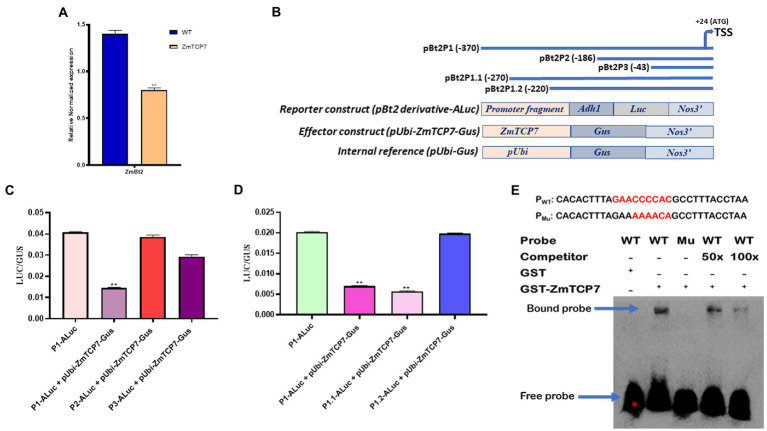
Transient overexpression analysis and identification of the binding site of ZmTCP7. **(A)** Trans-repression analysis of *ZmBt2* by transient overexpression of ZmTCP7 in maize endosperm protoplast cells by RT-qPCR; the bars indicate the mean ± SD of three technical replicates. Four independent experiments were performed and the results were found to be consistent in all cases. The significance of the difference between sample test and the control was analyzed by a two-sided *t*-test (^**^p < 0.001). **(B)** Schematic representations of *ZmBt2* deletion derivatives (upper panel), reporter, effector, and internal constructs (lower panel) for transient analysis of promoter activity. The deletion positions were indicated relative to the transcription start site (TSS) of the *ZmBt2* gene, *ZmTCP7* as an effector gene, the first intron of the maize *Adh1,* the Luc reporter gene, the Gus reporter gene and terminator Nos3 are all indicated; **(C,D)** Relative influence of ZmTCP7 on the activity of various derivatives of *ZmBt2* promoter deletion in the endosperm protoplasts of maize. The bars indicate the mean ± SD of three replicates. Results were found to be consistent in at least five independent experiments. The significance of the difference between each sample test and the control was analyzed by a two-sided *t*-test (^**^p < 0.001); **(E)** Electrophoretic mobility shift assay (EMSA) of the specific binding of ZmTCP7 to the *ZmBt2* promoter. EMSA analysis was performed with 30 bp (from −247 to −217) biotin-labeled probe (P*_WT_*) or biotin-labeled mutated probe (P*_Mu_*; upper panel) and unlabeled (competitor) oligonucleotides.

To characterize the transcriptional regulatory influence of ZmTCP7 on endosperm-specific *ZmBt2* gene expression, we transiently overexpressed ZmTCP7 in the MEPS and evaluated the expression of *ZmBt2* by RT-qPCR. Initially, we fused the entire ZmTCP7 coding sequence into an expression vector (pUbi-Gus) and generated the plasmid pUbi-ZmTCP7-Gus (the maize *ubiquitin* promoter drives the expression of ZmTCP7). Subsequently, we transiently transfected the construct pUbi-ZmTCP7-Gus into maize endosperm protoplasts and incubated for 24 h. The empty vector, pUbi-Gus, was also transiently introduced into protoplast cells and used as a control (WT). We then extracted total RNA from transfected cells and performed a RT-qPCR analysis after reverse transcription. Maize *actin* was used as the reference gene. The expression of *ZmBt2* was significantly suppressed by 42.86% in endosperm protoplast cells overexpressing *ZmTCP7* compared to WT (Figure 4A). This result suggests that ZmTCP7 functions as a transcriptional repressor of *ZmBt2* expression in maize endosperm. Thus, ZmTCP7 may be involved in the transcriptional regulation of storage starch accumulation in maize endosperm by targeting the promoter of *ZmBt2*.

To exemplify the mechanism by which ZmTCP7 targets and regulates the *ZmBt2* promoter, we created two fragments of promoter deletions (p*Bt2*P2 and p*Bt2*P3: Figure 4B; upper panel) of p*Bt2*P1, the promoter fragment that was used in the Y1H screening system, and transiently analyzed their activities to determine the region that contains the core binding motif targeted by ZmTCP7. p*Bt2*P1 and its two derivatives (p*Bt2*P2 and p*Bt2*P3) were inserted into pzaS3-ALuc and generated p*Bt2*P1-ALuc, p*Bt2*P2-ALuc and p*Bt2*P3-ALuc, as reporter constructs (Figure 4B; lower panel). The reporter constructs, along with pUbi-ZmTCP7-Gus as effector plasmid and pUbi-Gus as internal reference (Figure 4B; lower panel), were transiently co-transfected into maize endosperm protoplasts for expression assay. After 24 h incubation, fluorogenic assay (Gus) and luciferase activity (Luc) were analyzed. As shown in Figure 4C, the Luc/Gus activity ratio of protoplasts transfected with p*Bt2*P1-ALuc + pUbi-ZmTCP7-Gus was significantly reduced by 64.47% compared to the Luc/Gus activity ratio of protoplast cells transfected with only p*Bt2*P1-ALuc (control). This result confirmed the transcriptional repression of *ZmBt2* expression by ZmTCP7 in the transient overexpression assay (Figure 4A). Interestingly, the repressive influence of ZmTCP7 was completely eliminated in the cells transfected with p*Bt2*P2-ALuc + pUbi-ZmTCP7-Gus (Figure 4C), indicating that the promoter region between p*Bt2*P1 (−370) and p*Bt2*P2 (−186), about 184 bp, is critical for the transcriptional suppression of *ZmBt2* by ZmTCP7.

To further narrow down the 184-bp fragment to a more specific region, we generated two additional derivatives (p*Bt2*P1.1-ALuc and p*Bt2*P1.2-ALuc) of p*Bt2*P1-ALuc (Figure 3B; upper panel) with sequence deletions within the 184-bp fragment. We then performed a transient expression assay in MEPS and determined the Gus and Luc activity. The results showed that the deletion to −220 (p*Bt2*P1.2-ALuc) completely abolished the inhibitory effect of ZmTCP7 on the *ZmBt2* expression (Figure 4D), indicating that the *ZmBt2* promoter region between −270 to −220 (50 bp) contains the *cis*-elements targeted by ZmTCP7.

We then searched the entire 50-bp sequence (from −270 to −220) of the *ZmBt2* promoter for previously reported TCP consensus motifs: GGNCCCAC (for class I) and GTGGNCCC (for class II; Kosugi and Ohashi, 2002). Surprisingly, we identified a site “GAACCCCAC” at position −238 of the *ZmBt2* promoter (Supplementary Figure S3), which contained about six core elements “CCCCAC” synonymous with “NCCCAC” of the TCP class I consensus motif (GGNCCCAC). Therefore, we hypothesized that the binding motif for ZmTCP7 can be found at this site.

To ratify this hypothesis, we tested the binding specificity of ZmTCP7 with a 30-bp DNA sequence of the *ZmBt2* promoter region (from −247 to −218), which contains the putative binding site (GAACCCCAC; highlighted in red: Figure 4E; upper panel), in an electrophoretic mobility shift assay (EMSA). We first subjected the 30 bp DNA to a 3’end biotin labeling and subsequently used it as a probe (P*_WT_*) in EMSA. Then, we mutated the six-core elements (CCCCAC) in the WT and subjected it to a 3′-end biotin labeling to produce mutant probe (P*_MU_*: mutated elements are highlighted in red: Figure 4E; upper panel) and was used as a control in the EMSA. We also synthesized an unlabeled probe with the WT 30-bp oligonucleotide and used it for a competitor test. Subsequently, we incubated a bacterially expressed recombinant GST-ZmTCP7 fusion protein with the oligonucleotide probes. As shown in Figure 4E (lower panel), we detected a band shift when the GST-ZmTCP7 fusion protein was incubated with P*_WT_* but was eliminated in P*_MU_*, indicating that ZmTCP7 specifically binds to the *ZmBt2* promoter at the −238 site (GAACCCCAC). Additionally, we performed a competition test using unlabeled DNA as a competitor. Interestingly, the intensity of binding was considerably reduced with 50 times of competitor addition and almost completely removed when the amount of competitor was increased 100 times (Figure 4E; lower panel). These results indicate that ZmTCP7 specifically bind to the GAACCCCAC site in the *ZmBt2* promoter.

### ZmTCP7 overexpression suppresses *ZmBt2* transcription and causes transcriptional adjustment to expression of genes involved in endosperm starch biosynthesis of transgenic maize

**Figure 5:**
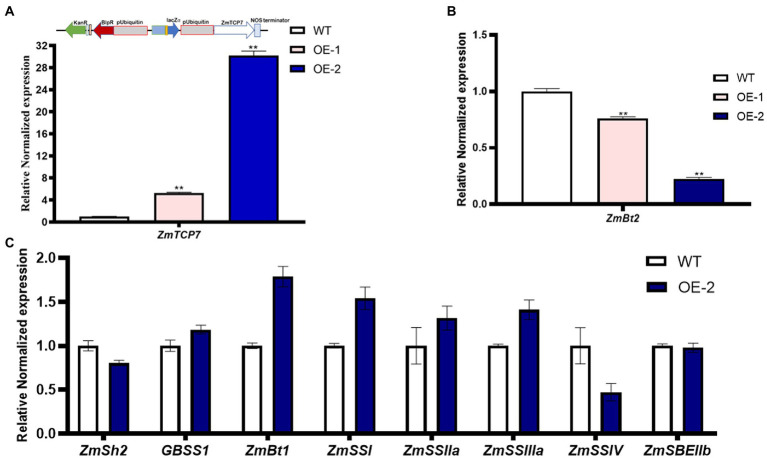
Transgenic overexpression analysis of ZmTCP7. **(A)** Schematic representation of the overexpression construct (upper panel) and the transcript levels of *ZmTCP7* (lower panel) in the endosperms from kernels of the transgenic overexpression (OE) lines and non-transformed (WT) kernels from the same ear by RT-qPCR. **(B)** The *ZmBt2* expression levels in the endosperm from kernels of the transgenic OE lines and WT by RT-qPCR assay. The significance of the difference between each transgenic OE line and WT was analyzed using a two-sided t-test (^**^p < 0.001); **(C)** Analysis of the expression of genes related to starch biosynthesis in endosperms from kernels of transgenic OE and WT by RT-qPCR analysis. The bars indicate the mean ± SD of three replicates. At least three independent experiments were performed and results were found to be consistent in all cases.

To further explore the functional role of ZmTCP7 in starch biosynthesis, we constructed an overexpression plasmid, pUB3301-eGus-ZmTCP7 (Figure 5A; upper panel), and integrated it into the genetic background of the maize B104 inbred line. The immature embryos derived from the developing ear of the maize B104 inbred line were transformed by the *Agrobacterium*-mediated transformation system and regenerated via tissue culture. The seedlings of the regenerated plants were transplanted to the field and managed under standard conditions. The bar strip test was used to identify positive transgenic plants in the seedling stage. Furthermore, PCR analysis was performed after transgenic plant genotyping by bar strip test as a further verification test (Supplementary Figure S4). We obtained two independent maize transgenic overexpression (OE) lines and were backcrossed with the WT to obtain T_0_ kernels. Subsequently, we performed RT-qPCR to analyze the level of the ZmTCP7 transcript in developing endosperms from T_0_ of the two OE lines sampled at 16 DAP. The level of ZmTCP7 transcript was significantly higher (*p < 0.01*) by 6 and 30 times in the endosperms of the two OE lines (OE-1 and OE-2, respectively) compared to the endosperms of WT (Figure 5A; lower panel). To further validate the transcriptional repression of *ZmBt2* expression by ZmTCP7, we analyzed the expression level of *ZmBt2* in the 16 DAP endosperms of the two independent transgenic OE lines. As shown in Figure 5B, *ZmBt2* expression levels in the endosperms of the OE-1 and OE-2 lines were significantly reduced by 23.91% and 77.58%, respectively, compared to WT. This result is consistent with the result of the transient overexpression assay in maize protoplasts (Figure 4A). Furthermore, the level of overexpression of *ZmTCP7* is consistent with the degree of repression of the *ZmBt2* transcript in the two OE lines (Figure 5A; lower panel and Figure 5B).

To further explore the transcriptional regulatory module of ZmTCP7 in the starch biosynthesis pathway, we examined the abundance of 8 genes related to starch synthesis in the 16 DAP endosperms of OE-2 and WT by RT-qPCR. Compared to WT, the expression levels of *ZmGBSS1, ZmBt1*, *ZmSS1*, *ZmSS2a,* and *ZmSS3a* were upregulated, although those of *ZmGBSS1* and *ZmSS2a* only changed slightly, in the OE-2 endosperm (Figure 5C). Whereas, *ZmSh2* and *ZmSSIV* transcript levels were downregulated, but the expression level of *ZmSBEIIb* remained unchanged in the endosperm of transgenic OE-2 as compared with the WT (Figure 5C). Similar transcriptional adjustments in the expression of these starch biosynthesis-related genes were also observed in maize endosperm protoplasts transiently overexpressing *ZmTCP7* (Supplementary Figure S5). These results showed that the relative expression levels of these genes were influenced by *ZmTCP7* overexpression, a possible indication that ZmTCP7 may be involved in the transcriptional regulation of different genes related to starch synthesis.

### ZmTCP7 overexpression lowers AGPase activity and reduces endosperm starch accumulation via inhibition of *ZmBt2* expression in transgenic maize

**Figure 6:**
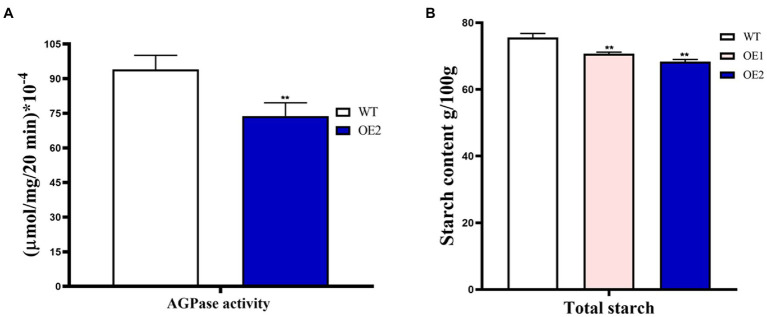
AGPase enzyme activity and starch content analyses of T_2_ transgenic maize overexpressing *ZmTCP7*. **(A)** *In vitro* analysis of the AGPase activity of endosperms of transgenic overexpression (OE) line and WT. Developing endosperms at 20 DAP from ears of at least 10 plants were used for AGPase activity; **(B)** Analysis of starch content in endosperms from mature kernels from OE lines and WT. Kernels from at least 10 ears were used for starch content analysis. At least three independent experiments were performed and results were found to be consistent in all cases. The significance of the difference between OE and the WT was analyzed by a two-sided *t*-test (^**^p < 0.001).

Since the *ZmBt2* gene encodes the small subunits of AGPase (an enzyme of the first committed step of starch synthesis in maize endosperm), we tested and compared the AGPase activity in the developing endosperms of WT and T_2_ transgenic OE-2 line at 20 DAP. Since AGPase activity progresses and is favored through rapid synthesis of ATP (Tuncel and Okita, 2013), we determined AGPase activity by evaluating ATP production *in vitro* using the Enhanced ATP Assay Kit (Beyotime, Beijing). Our results showed a significant 21.51% reduction in AGPase activity in the OE-2 endosperm compared to WT (Figure 6A). The result evidently indicated that overexpression of *ZmTCP7* in maize reduces AGPase activity by downregulating *ZmBt2* expression. To further elucidate the molecular pathway of ZmTCP7 in endosperm starch biosynthesis, we assayed the total starch content of the mature kernels of T_2_ transgenic OE lines. Interestingly, the total starch in 100 mg of mature kernel endosperm flour from OE-1 and OE-2 was significantly reduced by 6.50% and 9.58%, respectively, compared to 100 mg of mature kernel WT endosperm flour, which contained 75.59652 ± 0.4152 mg of starch (Figure 6B). Thus, the transcriptional repression of *ZmBt2* by ZmTCP7 resulted in significant reduction in AGPase enzyme activity and endosperm starch content of the transgenic maize. This suggests that ZmTCP7 plays an important regulatory role in determining the final level of starch in maize endosperm. Overall, our data suggest that ZmTCP7 functions as a negative regulator of endosperm starch accumulation by targeting and downregulating the expression of the endosperm-specific *ZmBt2* gene in maize.

## Discussion

More than 30 genes have been implicated in starch biosynthesis and accumulation in maize, and their functions have been clarified in great detail using loss and gain of function genetic approaches (Cossegal et al., 2008; López-González et al., 2019; Olsen, 2020). The maize endosperm-specific *ZmBt2*, which encodes the small subunits of AGPase, has always been one of the favorite gene models of geneticists, because of its role in the rate-limiting step of the starch biosynthetic pathway. Mutation or inactivation of *ZmBt2* has been associated with low AGPase activity, reduced accumulation of endosperm starch, shrunken/collapsed kernel phenotype, and significant modification of the carbohydrate metabolic pathway in seed endosperm (Hannah and Nelson, 1976; Preiss et al., 1990; Cossegal et al., 2008; Zhang et al., 2019).

The identification and characterization of all TFs that regulate *ZmBt2* expression will guide the future improvement program to modify the starch content of the maize endosperm in a planned way and will contribute to the overall increase in grain yield and quality. Previous studies suggest that the spatial and temporal expression of *ZmBt2* is largely governed by several TFs as its promoter contains multiple *cis*-acting regulatory elements (Li et al., 2018; Zhang et al., 2019; Hu et al., 2021). The Y1H screening system has been widely used to identify new TFs that target and interact with a given promoter bait sequence of a gene of interest (Sun et al., 2017). Here, we employed a highly efficient Y1H screening assay and successfully identify a novel ZmTCP7 TF that interacts with the recently characterized functional fragment of *ZmBt2* promoter. As noted in the result section, ZmTCP7 belongs to the TCP TF family, which share a highly conserved non-canonical bHLH structural motif (TCP domain): a domain that has been implicated in specific DNA binding activity and protein dimerization (Cubas et al., 1999; Kosugi and Ohashi, 2002). Although, several TCP TFs have been characterized as regulators of multiple developmental processes in higher plants, up till now, no member of this unique plant TF family has been associated with starch biosynthetic pathway; a major developmental event of the seed endosperm which determines the final grain yield and quality in cereal crops (Olsen and Becraft, 2013; Olsen, 2020). This study will be the first to throw new light on the molecular regulatory path of TCP TFs in angiosperms, as we characterize the molecular regulatory action of ZmTCP7 in starch biosynthetic pathway of maize endosperm.

Subcellular localization and expression analyzes show that ZmTCP7 is a nuclear protein that is predominantly expressed in developing endosperm. Interestingly, the abundance of the ZmTCP7 transcript accumulates in seed endosperm during its development from 10 to 25 DAP. The study by Hu et al. (2021) shows that this period (10 to 25 DAP) of endosperm development coincides with the time of dynamic changes in the endosperm transcriptome, in which complex modifications in the expression pattern of key genes related to starch synthesis occur in maize. Moreover, starch accumulation profiling further revealed that rapid accumulation of endosperm starch occurs around 10–20 DAP in maize (Hu et al., 2021). Therefore, the high level of ZmTCP7 expression in maize endosperm during this period suggests its regulatory role in the starch biosynthesis pathway.

Like most class I and class II TCPs, transactivation analysis showed that ZmTCP7 lacks transactivation activity in yeast system (Kosugi and Ohashi, 2002; González-Grandío and Cubas, 2016). Therefore, we assumed that ZmTCP7 may dimerize with other regulatory protein(s) or TF(s) to trans-repress *ZmBt2*, a speculation that remains to be investigated in future studies. Transactivation domain has been shown not to be conserved among TCPs genes (González-Grandío and Cubas, 2016). This possibly explain why TCP proteins dimerize and participate in multimeric regulatory module as observed in certain members of *Arabidopsis* TCP genes (Martín-Trillo and Cubas, 2010; Manassero et al., 2013). It will be expedient to explore the regulatory networks of *ZmBt2* by ZmTCP7 and its interactors in future studies to improve our understanding of its molecular action in endosperm development.

The fact that ZmTCP7 is evolutionally a class II TCP protein and is not self-activating (as shown in our data: Supplementary Figure S1 and Figure 3D) makes us hypothesize that ZmTCP7 may function as a transcriptional repressor of *ZmBt2* and a negative regulator of starch accumulation in maize endosperm. As expected, transient overexpression of *ZmTCP7* in maize endosperm protoplasts suppressed *ZmBt2* expression, suggesting that ZmTCP7 functions as a negative regulator of the rate-limiting step of the starch biosynthetic pathway. This result further reinforced the growing opinion that class II TCP genes are a repressor of plant development pathways, although recent evidence also indicates that some class II TCP can also act as a promoter of plant development (González-Grandío and Cubas, 2016; Sarvepalli and Nath, 2018; Ding et al., 2019). For example, maize ZmTB1 (ZmTCP1), one of the first four characterized members of TCP TF family, is a class II TCP gene that represses the growth of lateral branching and auxiliary organs (Cubas et al., 1999; Dong et al., 2019), whereas, another TCP class II maize gene, ZmTCP42 (GRMZM2G180568) was recently characterized as a positive regulator of drought stress (Ding et al., 2019), indicative of functional diversity among the TCP genes.

Analysis of the molecular mechanism by which ZmTCP7 interacts with *ZmBt2* promoter revealed that ZmTCP7 targets GAACCCCAC site of the *ZmBt2* promoter to govern its expression. The binding site (GAACCCCAC) of ZmTCP7 is highly similar to the consensus motif (GGNCCCAC) for class I TCPs (Kosugi and Ohashi, 2002). This is somewhat surprising because ZmTCP7, a class II (CIN clade) TCP gene, shows an affinity for a site similar to the binding site for class I TCPs. Researchers have insinuated that overlap of the binding motif by class I and class II TCPs indicates a shared regulatory pathway and could raise the possibility of competition or cooperation between TCP genes (González-Grandío and Cubas, 2016; Lucero et al., 2017). This may indicate that ZmTCP7 functions antagonistically or cooperatively with another class I TCP gene in maize that also targets the GAACCCCAC site of the *ZmBt2* promoter. This hypothesis requires future evaluation.

Since modification of a TF often induces phenotypic changes in plants (Viola and Gonzalez, 2016), we generated transgenic maize lines that overexpress ZmTCP7, to further explore the function of this novel TF. Consistent with the transient expression assay, transgenic overexpression of *ZmTCP7* reduced the transcript level of *ZmBt2* by ~77.58%. This considerable down-regulation of *ZmBt2* resulted in a 21.50% decrease in AGPase activity, a rate-limiting enzyme of the starch biosynthesis pathway, and consequently caused a substantial reduction (about 9.58%) in the endosperm starch content of mature kernels of transgenic maize relative to WT. Since *ZmBt2* encodes the small subunits of AGPase, transcriptional repression of the *ZmBt2* transcript by ZmTCP7 provides a strong basis for the significant decrease in the catalytic activity of AGPase and the substantial reduction in endosperm starch content of transgenic maize overexpressing *ZmTCP7*. By implication ZmTCP7 acts as a negative regulator of starch biosynthesis pathway via transcriptional regulation of *ZmBt2*. It is possible that ZmTCP7 takes part in other significant development processes in maize endosperm, thus, inactivating it might not be the best strategy. However, precise modification of the ZmTCP7 binding site via genome-editing technology could be a feasible approach to interrupt its binding specificity to *ZmBt2* promoter, and therefore could offer the potential to improve starch accumulation in maize endosperm.

Maize *Bt2* is a key gene of the starch biosynthesis pathway, and its promoter activity is strongly governed by transcriptional regulation that requires many TFs for transactivation due to the presence of multiple regulatory motifs within its promoter region (Li et al., 2018). In a recent study, two functionally redundant NAC TFs, ZmNAC128 and ZmNAC130, were identified and characterized as positive regulators of *ZmBt2* expression and starch biosynthesis in maize (Zhang et al., 2019). Surprisingly, these two functionally redundant TFs (NAC128 and NAC130) were found to target an ACGCAA site at position −66 upstream of the TSS (Zhang et al., 2019) within the regulatory region of the *ZmBt2* promoter, which is about 163 bp from the ZmTCP7 binding motif (GAACCCCAC; position −238 to −230). Currently, these two NAC proteins are the only TFs that have been reported so far as regulators that target *ZmBt2* for the regulation of starch biosynthesis. Therefore, the identification and functional characterization of the new ZmTCP7 TF in our current study offered new information on the molecular transcriptional regulation of *ZmBt2* expression and starch biosynthesis in maize.

Although our study has established that ZmTCP7 TF functions as a negative regulator of starch biosynthesis, the mechanism that underscores the biological importance of its role remains to be elucidated in future research. Several factors including endosperm cell sucrose level, phytohormones such as abscisic acid (ABA), heat and dehydration stress have been shown to mediate the regulation of AGPase activity and expression of its encoding genes (*Bt2* and *Sh2*) and starch metabolism in maize endosperm (Huang et al., 2016; Li et al., 2018; Yang et al., 2018).

A higher amount of sucrose activates the activity of AGPase and the expression of its encoding genes (*Bt2* and *Sh2*), and improves starch accumulation in endosperm tissue (Tuncel and Okita, 2013; Li et al., 2018). In contrast, heat or dehydration stress causes a considerable reduction in activities of enzymes and expression of genes associated with starch biosynthesis (Yang et al., 2018). Accumulation of ABA in cereal kernels has been shown to promote activity of enzymes and expression of genes related to starch biosynthesis, as well as facilitate sucrose transport into endosperm cytosol and mediates response to heat and dehydration stress (Maestri et al., 2002; Chen et al., 2011; Li et al., 2018). Evidently, ABA plays an important role in the regulation of starch biosynthesis and the expression of genes related to the pathway, as well as in the physiological modulation of environmental stress responses. However, growing evidences revealed that ABA alone is not sufficient but generally functions synergistically in combination with sucrose to mediate starch biosynthesis regulation (Chen et al., 2011; Huang et al., 2016; Li et al., 2018).

Interestingly, the functional fragment of *ZmBt2* promoter (DNA bait sequence used in Y1H screening assay for ZmTCP7 identification in this study), which was identified by Li et al. (2018), contains two adjacent regions, −370/−186 (sucrose-responsive) and −186/−43 (ABA-responsive), which are synergistically responsive to a combination of sucrose and ABA signals. As a negative regulator, we speculated that regulation of starch biosynthesis by ZmTCP7 may be mediated by synergistic action of combination of sucrose and ABA in response to environmental stress signals. Surprisingly, the ZmTCP7 binding site (GAACCCCAC) is located within the promoter region that is sensitive to sucrose. Therefore, it is possible that ZmTCP7 interacts with other factors that target the ABA-responsive region of the *ZmBt2* promoter to cooperatively regulate starch biosynthesis in response to an inducible stress signal. This notion is logical, as ZmTCP7 binding site (GAACCCCAC) is in close proximity with different putative *cis*-acting elements (Supplementary Figure S3), including I-box (about 51-bp to the ZmTCP7 binding site), Skn-1 motif (about 16-bp to the ZmTCP7 binding site), and ABA-response element (G-box: CACGTG; which is about 98-bp from the ZmTCP7 binding site), a highly conserved motif for the regulation of various environmental and physiological signals (Williams et al., 1992). We suggest future research effort to identify and characterize factors that interact and function cooperatively with ZmTCP7 to transcriptionally regulate *ZmBt2*, and unravel the molecular mechanism underscoring the biological significance of ZmTCP7 role in endosperm development. Such investigations will improve our understanding of the basis for the molecular action of ZmTCP7 and will also guide the genome modification strategy to improve the endosperm starch content in maize.

## Data availability statement

The original contributions presented in the study are included in the article/Supplementary material, further inquiries can be directed to the corresponding authors.

## Author contributions

BSA: methodology, investigation, data validation formal analysis, visualization, and writing—original draft, review and editing. YL: conceptualization, supervision, project administration, and writing—review. YW, CD, and LG: investigation and validation. HL, GY, and JZ: resources. YHuang and YHu: supervision, writing—review, project administration, and funding acquisition. All authors contributed to the article and approved the submitted version.

## Funding

This work was supported by the National Key Research and Development Program of China (2021YFF1000304) and the National Natural Science Foundation of China (31971960).

## Conflict of interest

The authors declare that the research was conducted in the absence of any commercial or financial relationship that could be construed as a potential conflict of interest.

## Publisher’s note

All claims expressed in this article are solely those of the authors and do not necessarily represent those of their affiliated organizations, or those of the publisher, the editors and the reviewers. Any product that may be evaluated in this article, or claim that may be made by its manufacturer, is not guaranteed or endorsed by the publisher.

## Footnotes

^1^ https://ensembl.gramene.org/Zea_mays/Tools/Blast

^2^ https://maizegdb.org

^3^ https://www.grassius.org/index.php

^4^ http://datf.cbi.pku.edu.cn

^5^ https://maizegdb.org
