# Supplementary material for: The novel ZmTCP7 transcription factor targets AGPase-encoding gene ZmBt2 to regulate storage starch accumulation in maize
Source: Front Plant Sci. 2022 Jul 15;13:943050. doi: 10.3389/fpls.2022.943050 (PMC9335043; doi:10.3389/fpls.2022.943050)
Supplement: Supplementary file 1 [file Data_Sheet_1.pdf]

Supporting information for

The novel ZmTCP7 transcription factor targets AGPase-encoding gene *ZmBt2* to regulate storage starch accumulation in maize

Babatope Samuel Ajayo, Yangping Li, Yayun Wang, Chengdong Dai, Lei Gao, Hanmei Liu, Guowu Yu, Junjie Zhang, Yubi Huang *, Yufeng Hu *.

*Correspondence: yubihuang@sohu.com; huyufeng@sicau.edu.cn

This file includes:

Figure S1: Phylogenetic tree of maize, rice, and *Arabidopsis* TCP TF family.

Figure S2: Sequence alignment of ZmTCP7 (GRMZM2G035944) and ZmTCP23 (GRMZM2G120151).

Figure S3: DNA sequence of the functional region of *ZmBt2* promoter showing the binding site of ZmTCP7.

Figure S4: Generation of transgenic maize plants.

Figure S5: Analysis of the expression of genes related to starch biosynthesis in maize endosperm protoplasts transiently overexpressing ZmTCP7.

Table S1: Primer sequences for RT-qPCR and amplification of DNA fragments used in different constructs.

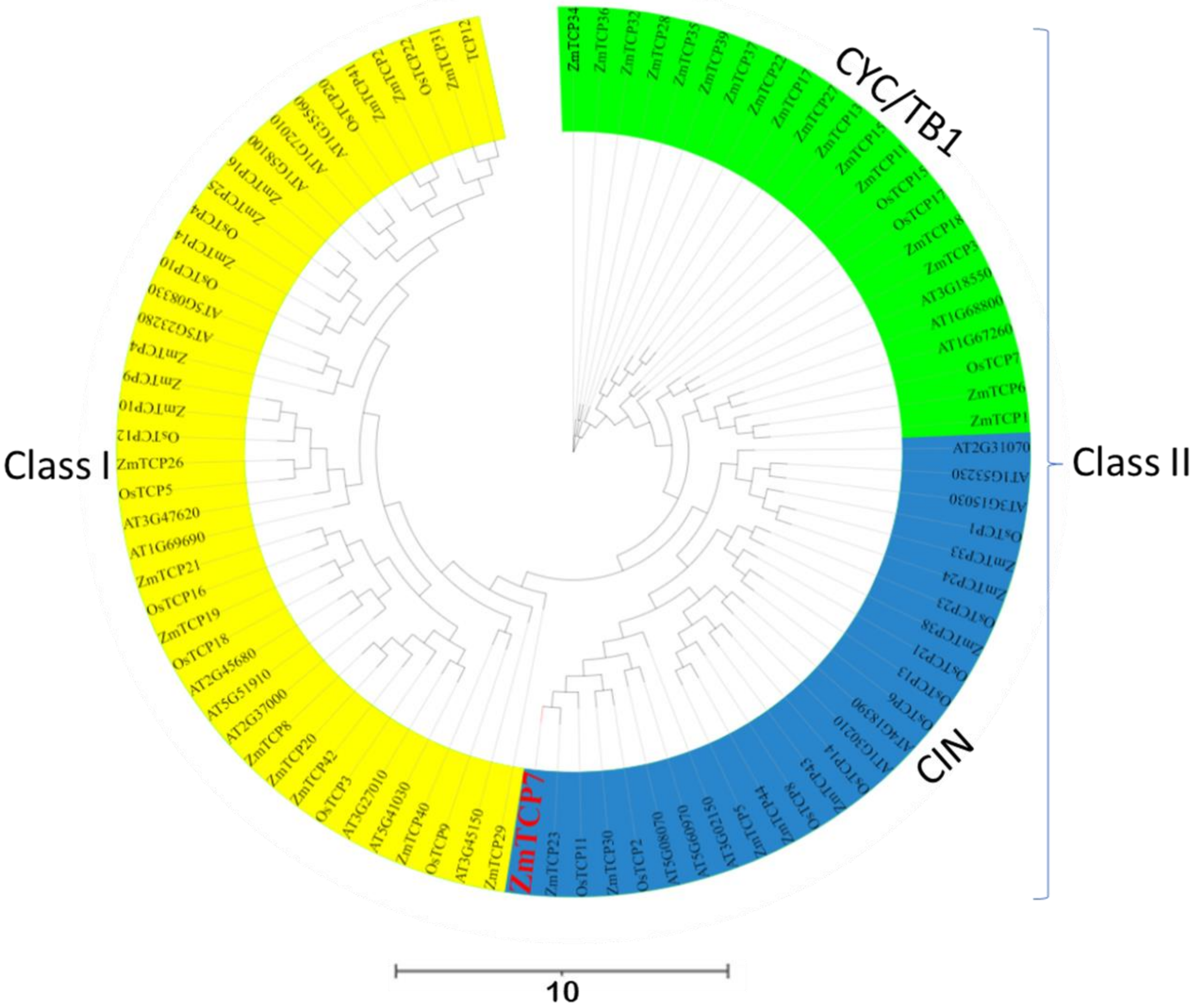

Figure S1: Phylogenetic tree of maize, rice, and *Arabidopsis* TCP TF family. The class I, CIN and CYC/TB1 subdivisions of class II TCPs, are highlighted in yellow, blue and green, respectively. ZmTCP7 of the CIN clade is indicated in red. Multiple alignment of the protein sequences of the TCP TFs in the various crops was performed using MEGA X, and the phylogenetic tree was constructed using the neighbor joining method with 1000 bootstrap replicas (Saitou and Nei, 1987; Kumar et al., 2018). Evolutionary distances were computed using the JTT matrix-based method (Jones et al., 1992). Interactive Tree of Life (iTOL) online tool was used to produce the final display and annotation of the tree (Letunic and Bork, 2021).

```
          78.3% identity in 304 residues overlap; Score: 944.0; Gap frequency: 12.2%

 ZmTCP7   B4FHV6|B4F     1 MISGNLTNEDLGAGR-TKAEGPTTAARA---VAKSPVLPSSRHWPPSTESRIVRVSRVFG
ZmTCP23   C0HEC8|C0H     1 MISGNLNNEELGGGGCTKAEGPGAAGGAGAVVAKSPVLSSSRHWPPSTESRIVRVSRVFG
                           ****** ** ** *  ******  *  *   ******* *********************

          B4FHV6|B4F    57 GKDRHSKVRTVKGLRDRRVRLSVPTAIQLYDLQDRLGLSQPSKVVDWLLDAAQHEIDKLP
          C0HEC8|C0H    61 GKDRHSKVRTVKGLRDRRVRLSVPTAIQLYDLQDRLGLSQPSKVVDWLLDAAQDEIDKLP
                           ***************************************************** ******

          B4FHV6|B4F   117 PLQFPPQAAQDLVAHLPPPSMVAPFA---ADRAEA------------DGDKRHCHAGVKG
          C0HEC8|C0H   121 PLQFPPQG-QDLVAHLPP-SMVAPFANVAADRAAAAAAAANASAAMAEGGKRHCHGGIKG
                           *******  ********* *******   **** *             * ***** * **

          B4FHV6|B4F   162 LMG--LNNSIRLVNATMPLAHGLYYTPGESWTGTNADDAAAHDHQ---------TVAHGH
          C0HEC8|C0H   179 LMGMGLNNSIGLANATMPLAHGLYYTPGESW--TNGGNAAVHDHQVSHGGTSPQTVAH-H
                           ***  ***** * ******************  **   ** ****         **** *

          B4FHV6|B4F   211 SPFSSLLSLAAPGPQLVFYSSEGGGFTMKEEATGYQFPVQNSLDHSQGQLSLSSARSFLR
          C0HEC8|C0H   236 SPFSSLLSLAAPGPQLVFYSPEGGGFAMKE-ATDHQFAV-DSLDHSQGQLSLSSARSFLH
                           ******************** ***** *** **  ** *  ******************

          B4FHV6|B4F   271 PGNQ
          C0HEC8|C0H   294 PGNQ
                           ****
```

Figure S2: Sequence alignment of ZmTCP7 (B4FHV6|B4F) and ZmTCP23 (C0HEC8|C0H). SIM – Alignment Tool for protein sequences (https://web.expasy.org/sim/) was used to perform the alignment.

TTGGAACAGCAAGCTATACAGCGCTATATAACAAACTTGCACAATAATATGCTACATTTCTGAAAGCAAACTCCGC
-370
TTATCTTCGCGTTAAGGAATGGTATTACACTTAAAATGACAAAATATCACACTTTAGAACCCCACGCCTTTACCTAA
-238 ~ -230 (Binding site)
AAAAAAGTTGATTGATACAAGAACAAATGTTTACAGCTTGTTACACCTCCCATCAAGCAAAGAAATGGAATCTTTT

ATTTTATGTACACGTGTACGGGTAGCTATTGTTTATAAATTGCAGAAGACACCTCAACATGATGGATAATTGTATAC

GCAAAACTACTTTCCTCGAATTCCCCATTCTATAAATTCCAGTTAAACTTAATTCGTAAGATCAATCATAATTCTCGA
+1
GTTGCAAACC

Figure S3: DNA sequence of the functional region of *ZmBt2* promoter showing the binding site of ZmTCP7 (GAACCCCAC: -238 ~ -230). Putative motifs that are in close proximity to the ZmTCP7 binding site are indicated in color: green, I-box motif; yellow, Skn-1 motif; pink, G-box motif.

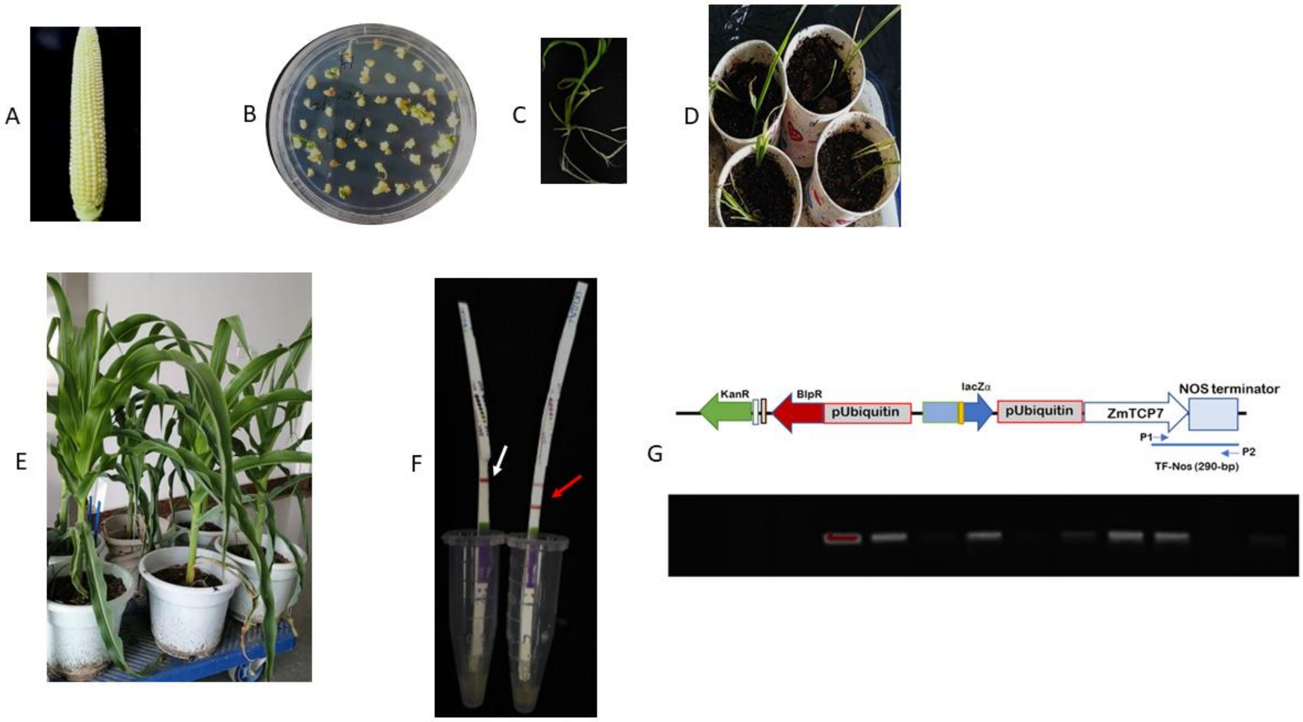

Figure S4: Generation of transgenic maize plants. **A**, developing ear at 10 DAP; **B**, callus culture with growing calli; **C**, putative transgenic plantlet obtained from successful regeneration; **D**, hardening of putative transgenic plantlets in small pots containing peat mose before transplanting; **E**, hardened regenerated plants growing in pots containing loam soil; **F**, Bar strip test results showing negative (single bar; indicated by the white arrow head) and positive transgenic (double bar; indicated by the red arrow head) plants. The bar strip test was used in the identification of positive transgenic plants and kernels; **G**, the schematic representation of the overexpression construct (upper panel) and genotyping of the positive transgenic plants via PCR analysis to confirm the bar strip test results. The amplification region designated as TF-Nos (290-bp) is indicated by the blue line spanning the primer pairs, P1 and P2, as shown in the overexpression construct.

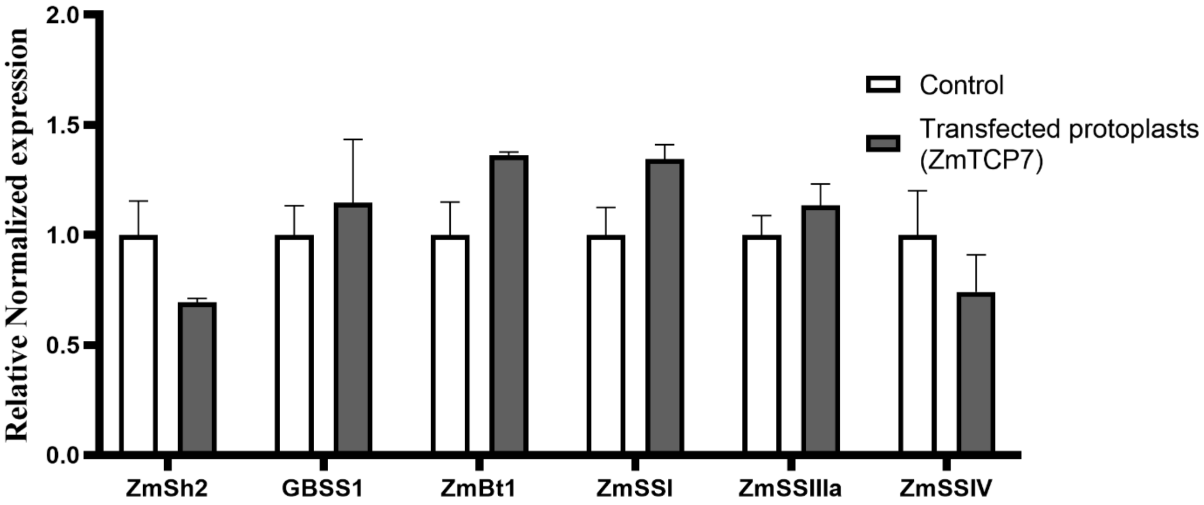

Figure S5: Analysis of the expression of genes related to starch biosynthesis in maize endosperm protoplasts transiently overexpressing ZmTCP7 by RT-qPCR. The bars indicate the mean ± SD of three replicates.

Table S1: List of primers used in this study. The restriction enzyme sites are underlined.

Primer name	Primer sequence (5’ to 3’)	Notes
pBt2P1-F-H*ind*III	AAGCTTTTGGAACAGCAAGCTATACAG	pAbAi construct
pBt2P-R-X*ho*I	CTCGAGGGTTTGCAACTCGAGAATTATG	
pBt2P1-F-P*st*I	CTGCAGTTGGAACAGCAAGCTATACAG	Promoter deletion constructs
pBt2P1.1-F- P*st*I	CTGCAGATCTTCGCGTTAAGGAATGGTA	
pBt2P1.2-F-P*st*I	CTGCAGTAAAAAAAAGTTGATTGATACAAGAAC	
pBt2P2-F-P*st*I	CTGCAGTACAGCTTGTTACACCTCCCAT	
pBt2P3-F-P*st*I	CTGCAGTCCCCATTCTATAAATTCCAG	
pBt2P-R-B*amH*I	GGATCCGGTTTGCAACTCGAGAATTATG	
ZmTCP7-F	ATGATAAGCGGCAACCTCACCAATGAAG	Nested PCR
ZmTCP7-R	GAGCAGTAGAGATCAGTGACCTAATC	
pGADT7-Rec / ZmTCP7-F-N*de*I	CATATGATGATAAGCGGCAACCTCACCAATGAAG	pGADT7-Rec construct
pGADT7-Rec / ZmTCP7-R-B*amH*I	GGATCCTCATACCTGGTTGCCGGGGC	
pGBKT7 / ZmTCP7-F-E*coR*I	GAATTCATGATAAGCGGCAACCTCACCAATGAAG	pGBKT7-Rec construct
pGBKT7 / ZmTCP7-R-B*amH*I	GGATCCTCATACCTGGTTGCCGGGGC	
eGFP-ZmTCP7-F-K*pn*I	GGTACCATGATAAGCGGCAACCTCACCAATGAAG	pCAMBIA2300-35S-eGFP construct
eGFP-ZmTCP7-R-X*ba*I	TCTAGATCATACCTGGTTGCCGGGGC	
pUbi-ZmTCP7-Gus-F-B*amH*I	GGATCCATGATAAGCGGCAACCTCACCAATGAAG	pUbi-Gus construct
pUbi-ZmTCP7-Gus-R-S*ac*I	GAGCTCTCATACCTGGTTGCCGGGGC	
pGST-ZmTCP7-F-E*coR*I	GAATTCATGATAAGCGGCAACCTCACCAATGAAG	pGEX-6-1 construct
pGST-ZmTCP7-R-X*ho*I	CTCGAGTCATACCTGGTTGCCGGGGC	
pUB3301-eGus-ZmTCP7-F-H*ind*III	AAGCTTTGCAGCGTGACCCGGTCG	pUB3301-eGus (overexpression) construct
pUB3301-eGus-ZmTCP7-R-P*mlI*	CACGTGTCATACCTGGTTGCCGGGGC	
AD-F	TAATACGACTCACTATAGGGCGAGC	Yeast colony PCR
AD-R	AGATGGTGCACGATGCACAG	
ZmTCP7-F	CTTCTCATCTCTGCTGTCTCTTG	RT-qPCR
ZmTCP7-R	GATAACCGGTGGCTTCTTCTT	
ZmBt2-F	ACTAATGGGTGCGGACTACTATG	
ZmBt2-R	TACCCGTCTGTCTCCATTGC	
Actin-F	GCTACGAGATGCCTGATGGTC	
Actin-F	CCCCCACTGAGGACAACG	
ZmSh2-F	TGGGAGCGGACACCTATG	
ZmSh2-R	TCACCACGATTCCAGACCTT	
ZmBt1-F	GGTGTTCCAGTGGATCATG	
ZmBt1-R	CCGTGTCATAGGTGAAATG	
ZmGBSS1-F	GTCGAAGGCGAGGAGATC	
ZmGBSS1-R	CGCTTATTAGGTTGTGCCA	
ZmSS1-F	GTCTGCTTTGGCTGCCTTG	
ZmSS1-R	AGGACAACAACACAGGTAATAATC	
ZmSS2-F	GTTTCCATTCCCAATCCTGA	
ZmSS2-R	CGTCAGGCTCGTCAAAACC	
ZmSS3-F	TTGGAATCATCACTCGTCTAACAG	
ZmSS3-R	AACATAGCTTCACCCGGCCA	
ZmSS4-F	GGAGGTGCGAAATGGCTT	
ZmSS4-R	AGGGAACTGTCTCAATGTCTCTG	
ZmSBE2b-F	CGAAAGCCTGGGGTGTAT	
ZmSBE2b-R	CACTGGAGCATAGACGACACAT	
